# Case of Cancer of the Conjunctiva

**Published:** 1859-03

**Authors:** Charles Brackett

**Affiliations:** Rochester, Ind.


### ARTICLE IX.

### CASE OF CANCER OF THE CONJUNCTIVA. EXTIRPATION OF THE GLOBE OF THE EYE. CASE OF NASAL POLYPUS CURED BY OPERATION AND APPLICATIONS.

BY CHARLES BRACKETT, M.D., OF ROCHESTER, IND.

The following interesting case came under my observation in October, 1858. The subject, Abraham Blue, aged about seventy, wished me to examine his right eyė, which was protuberant, having the appearance and consistence of highly congested cerebral substance, secreting quite freely a gummy sort of pus, accompanied by slight lancinating pains, but most troublesome to the patient on account of the free discharge of pus from the surface.

I judged, from first sight, that it was a case of medullary sarcoma. The patient said that he had had, for several years, "what the doctors called pterygium," which had followed a severe blow on the right cheek, which had indented the mala bone. Said it had grown externally, and that he thought the internal eye was sound; that he could tell in which direction the light was.

I gave a wash of sul. zinc solved in water, and directed him to come again in one week; using, in the mean time, occasional laxative doses of sul. magnesia.

He returned, and I determined to try first to save the ball, as, by exploration with needle, I found it sound. Assisted by Dr. Gould, by a tedious operation, I removed the brain-like mass all clean from the conjunctiva, cauterized the surface with the nit. argt., and gave a saturated solut. to apply twice per day. After eight weeks he returned, the fungus mass larger than ever, so that the lids could not be closed over it. The same profuse discharge present as before; had been growing for four weeks. He wished to be freed from the disease; and, assisted by Drs. Gould and Wilkins, I again proceeded to explore. At this time the needle passed through the soft mass almost with no resistance, three-fourths of an inch, then encountered the ball, and passed through the coats of the ball almost as easily as through the medullary mass, proving that they were rapidly undergoing the softening metamorphosis, and declaring the speedy extirpation of the ball the only hope.

I told the patient the condition of things, and he cheerfully consented to part with the offending eye, telling me to "pluck it out."

After the removal, the coats were all softened, the retina congested, the lens either absorbed or lost, as I could not find it, the vitreous humor lessened in quantity and becoming clouded. The optic nerve, where cut, appeared healthy, as did all parts posterior to the ball.

Here is a fungous growth, commencing in or on the conjunctiva, protruding between the palpebræ; and the parts below undergoing transformation into like disease; a soft, brain-like mass, quite vascular, and, since the first operation, attended with constant pain, aggravated occasionally by severe lancinating pains. Was it *cancer cerebriform?* This is the conclusion I have arrived at.

The patient had been long troubled with dysuria. This left him from the time of removal of the ball, and has not yet reappeared. I thought that probably the dose of *morphine and soda* had something to do with the relief. At any rate, this was all the medicine he had taken, except the sul. mag. as a laxative.

The operation was performed on Wednesday, the 20th of December, and from that day on, the patient was up most of the time till the 26th, when he felt well enough to ride home, a distance of about twenty miles.

Lewis Spatz, aged twenty, with polypus of nose, came to me to have it removed. After some five or six different trials, succeeded in relieving him perfectly. The polypus was tough and fibrous, and filled the whole sup. post naris, and protruding from anterior nostril. The difficulties in the way of its complete removal were, profuse hemorrhage, and the shreddy condition of the mass after a part had been taken away. I used the ligature, knife and forceps, at different times. Upon finding I could not get it all after various trials, I applied sul. zinc and pulv. rad. sanguinaria. This so completely killed the whole mass, after a week's daily application, that I removed it entire with the forceps without difficulty, and with comparatively slight pain to the patient. The snuffing of pulv. sanguinaria, canadensis and sul. zinc, restored the mucous lining to complete health, now for nearly six months past. Here the blood root and zinc performed a valuable part by killing the whole mass, toughening it, and rendering its removal an *easy* matter, when, before, I was almost discouraged, as it seemed to grow faster than I could remove it. Like the heart of Prometheus, "Quantum vero interdin exederat, tantrim nocte cresebat."

This is one of the preparations of the *celebrated* American cancer doctor, in London, before whose *fell* hand the horrid cancer crawls away, and hides his head in grief. The discovery, he claims, he made during a long "imprisonment among the North-American Indians on the shores of Lake Superior," by the great "Gitchee Gummee, the big sea water," and our cousin Johnny Bull lops his ears before the "Cancer Master." Seriously the articles are both of vast utility, and probably not as fully appreciated by the profession generally as their virtues warrant, especially in the cure of nasal polypi. I have by them alone succeeded in at least two cases of soft polypi.

